# Hippocampal alterations in glutamatergic signaling during amyloid progression in AβPP/PS1 mice

**DOI:** 10.1038/s41598-020-71587-6

**Published:** 2020-09-02

**Authors:** Kevin N. Hascup, Caleigh A. Findley, Lindsey N. Sime, Erin R. Hascup

**Affiliations:** 1 Department of Neurology, Center for Alzheimer’s Disease and Related Disorders, Neurosciences Institute, Southern Illinois University School of Medicine, P.O. Box 19628, Springfield, IL 62794-9628 USA; 2 Department of Pharmacology, Southern Illinois University School of Medicine, Springfield, IL USA; 3 Department of Medical Microbiology, Immunology, and Cell Biology, Southern Illinois University School of Medicine, Springfield, IL USA

**Keywords:** Sensors and probes, Predictive markers, Alzheimer's disease, Dementia, Neurodegeneration

## Abstract

Our previous research demonstrated that soluble amyloid-β (Aβ)_42_, elicits presynaptic glutamate release. We hypothesized that accumulation and deposition of Aβ altered glutamatergic neurotransmission in a temporally and spatially dependent manner. To test this hypothesis, a glutamate selective microelectrode array (MEA) was used to monitor dentate (DG), CA3, and CA1 hippocampal extracellular glutamate levels in 2–4, 6–8, and 18–20 month-old male AβPP/PS1 and age-matched C57BL/6J control mice. Starting at 6 months of age, AβPP/PS1 basal glutamate levels are elevated in all three hippocampal subregions that becomes more pronounced at the oldest age group. Evoked glutamate release was elevated in all three age groups in the DG, but temporally delayed to 18–20 months in the CA3 of AβPP/PS1 mice. However, CA1 evoked glutamate release in AβPP/PS1 mice was elevated at 2–4 months of age and declined with age. Plaque deposition was anatomically aligned (but temporally delayed) with elevated glutamate levels; whereby accumulation was first observed in the CA1 and DG starting at 6–8 months that progressed throughout all hippocampal subregions by 18–20 months of age. The temporal hippocampal glutamate changes observed in this study may serve as a biomarker allowing for time point specific therapeutic interventions in Alzheimer’s disease patients.

## Introduction

Alzheimer’s disease (AD) is an age-related neurodegenerative disorder characterized by progressive anterograde amnesia, cerebral atrophy, functional decline, and eventual death. Available pharmacotherapy options either target cholinesterase inhibitors or partial antagonism of the N-methyl-D-aspartate receptors (NMDAR). However, these treatments have limited efficacy, are symptomatic, and are unable to decelerate disease progression^1,2^ possibly because they are administered at advanced AD stages when synapse loss is too pronounced. This lack of disease modifying treatment is due to poor identification of effective biomarkers for early diagnosis. Accordingly, intense efforts are ongoing to understand neurological changes associated with AD progression.

The hallmark proteinopathies associated with AD include extracellular β-amyloid (Aβ) plaques and intracellular hyperphosphorylated tau tangles that are both hypothesized to accumulate 20–30 years prior to onset of mild cognitive impairment (MCI) and eventual AD^3^. In humans, the deposition of these proteins typically follows a sequential pattern throughout the medial temporal lobe and thus is often used for post-mortem identification of disease severity^4^. Deposition is often first observed in the entorhinal cortex and CA1 followed by the dentate (DG) and CA3^5,6^. The accumulation of these proteins either coincides with, or causes, alterations in neurotransmitter dynamics^7^. For example, hippocampal hyperactivation during a memory encoding task was observed a decade before cognitive decline in a cohort of individuals with the presenilin 1 (PS1) E280A mutation that is associated with early onset AD^8^. A 3-year longitudinal study showed that elevated hippocampal activity was present in plaque positive MCI patients who subsequently showed faster progression of cognitive decline compared to MCI patients without plaque accumulation^9^. Finally, tau accumulation in cognitively normal older adults was associated with hippocampal hyperactivity^10,11^. These studies support that aberrant hippocampal neuronal activity precedes clinical AD diagnosis and suggests elevated glutamatergic signaling as the driving force associated with disease progression^12,13^.

Glutamate is prevalent in neocortical and hippocampal pyramidal neurons and plays a role in synaptic plasticity, learning, and memory consolidation. Synaptic glutamate signaling is tightly regulated by clearance through high-affinity excitatory amino acid transporters^14^. However, postmortem analysis shows that vesicular glutamate transporter 1 boutons were elevated in pre-clinical AD cases^15^ while glutamate transporters were decreased in AD patients^16^. Indicating mechanisms responsible for glutamatergic regulation are altered during different stages of disease progression. A contributing factor to this dysregulation is the accumulation of soluble Aβ isoforms that initiates synaptic dysfunction causing the eventual neurodegeneration^17,18^. For example, our laboratory and others have demonstrated that Aβ_42_ elicits glutamate release^19–21^. Accordingly, mouse models of progressive cerebral amyloidosis, such as the double transgenic mice expressing the amyloid precursor protein (Mo/HuAPP695swe) and Presenilin 1 (PS1-dE9) genes (AβPP/PS1), hippocampal glutamate is elevated at 12 months of age^22,23^. Therefore, understanding changes to glutamatergic signaling at different stages of AD progression could lead to the development of disease-stage specific therapeutics.

We hypothesized that accumulation and deposition of Aβ_42_ altered glutamatergic neurotransmission in a temporally and anatomically spatial dependent manner. To probe this, in vivo hippocampal glutamate dynamics were measured using an enzyme-based microelectrode array (MEA) coupled with constant potential amperometry in AβPP/PS1 mice. To determine the effects of disease progression on hippocampal glutamate, 2–4 (increased soluble Aβ_42_ but no plaque accumulation), 6–8 (initial plaque accumulation) and 18–20 (severe plaque accumulation) month old AβPP/PS1 mice were examined. Age-matched C57BL/6J mice were used as genetic background, normal aging controls. The results presented here indicate that AβPP/PS1 mice develop elevated basal and stimulus-evoked glutamate release before plaque accumulation. These elevated glutamate levels change with age and in a hippocampal subregion dependent manner that is independent from normal aging, which may allow for the development for more targeted therapeutics on an individual level.

## Results

### Learning and memory retrieval

**Figure 1:**
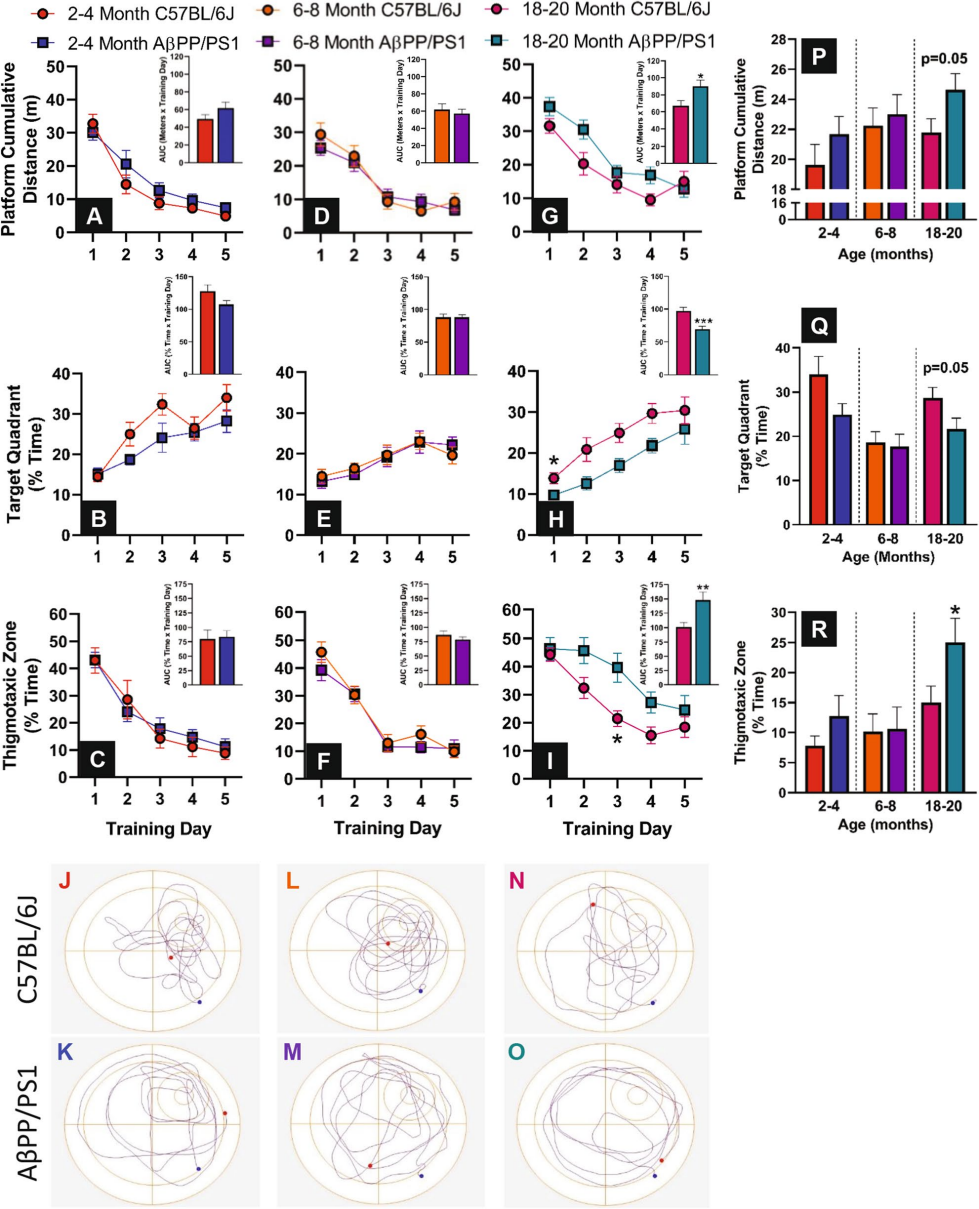
MWM learning and memory recall. During the 5 day MWM training, each day consisted of 3 trials that were averaged into a single data point for the cumulative distance (**A**,**D**,**G**), percentage of time in the target quadrant (**B**,**E**,**H**) and percentage of time in the thigmotaxic zone (**C**,**F**,**I**) for each age group. Line graphs were analyzed using a two-way ANOVA (Training Day × Genotype) with Sidak’s post-hoc analysis. The insets indicate the area under the curve for the five training sessions that were analyzed using a two-tailed t-test. Representative track plots of the 60 s probe challenge are shown for both genotypes at all ages tested (**J**–**O**). The blue and red dots indicate maze entrance and ending location, respectively. The cumulative distance from the platform (**P**), percentage of time in the target quadrant (**Q**) and percentage of time in the thigmotaxic zone (**R**). A two-tailed t-test was used to compare genotypes within age groups. *p < 0.05, **p < 0.01, C57BL/6J n = 10–14, AβPP/PS1 n = 10–13.

Cognitive performance was assessed using the Morris water maze (MWM) learning and memory recall behavioral paradigm. No differences in learning were observed between 2–4 month and 6–8 month old AβPP/PS1 and age-matched C57BL/6J mice (Fig. 1A–F). At the 18–20 month age range AβPP/PS1 were slower to learn the location of the hidden escape platform compared to age-matched C57BL/6J mice as indicated by the cumulative distance from the platform (F_1,24_ = 5.111, P = 0.33) and the area under the curve (AUC) of this parameter (t_24_ = 2.488, p = 0.02) as shown in Fig. 1G. This age group of AβPP/PS1 mice also spent less time searching the target quadrant for the escape platform (F_1,24_ = 13.10, P = 0.0014) over the five training sessions (t_24_ = 3.824, p = 0.0008 as shown in Fig. 1H. Additionally, AβPP/PS1 mice spent more time navigating the periphery of the maze during the training sessions as indicated by percentage of time in the thigmotaxic zone (F_1,24_ = 7.284, P = 0.01) and the AUC of this parameter (t_24_ = 3.016, p = 0.006) as shown in Fig. 1I. Representative probe challenge path traces are shown in Fig. 1J–O. No differences in memory recall were observed during the probe challenge between 2–4 and 6–8 month old AβPP/PS1 and C57BL/6J mice. However, at 18–20 months of age, AβPP/PS1 had a further cumulative distance from the former location of the hidden escape platform (Fig. 1P; t_24_ = 2.049, p = 0.05), searched less in the target quadrant (Fig. 1Q; t_24_ = 2.036, p = 0.05), and more time searching the periphery of the pool (Fig. 1R; t_24_ = 2.145, p = 0.04) than age-matched C57BL/6J mice.

### In vivo DG glutamate dynamics

**Figure 2:**
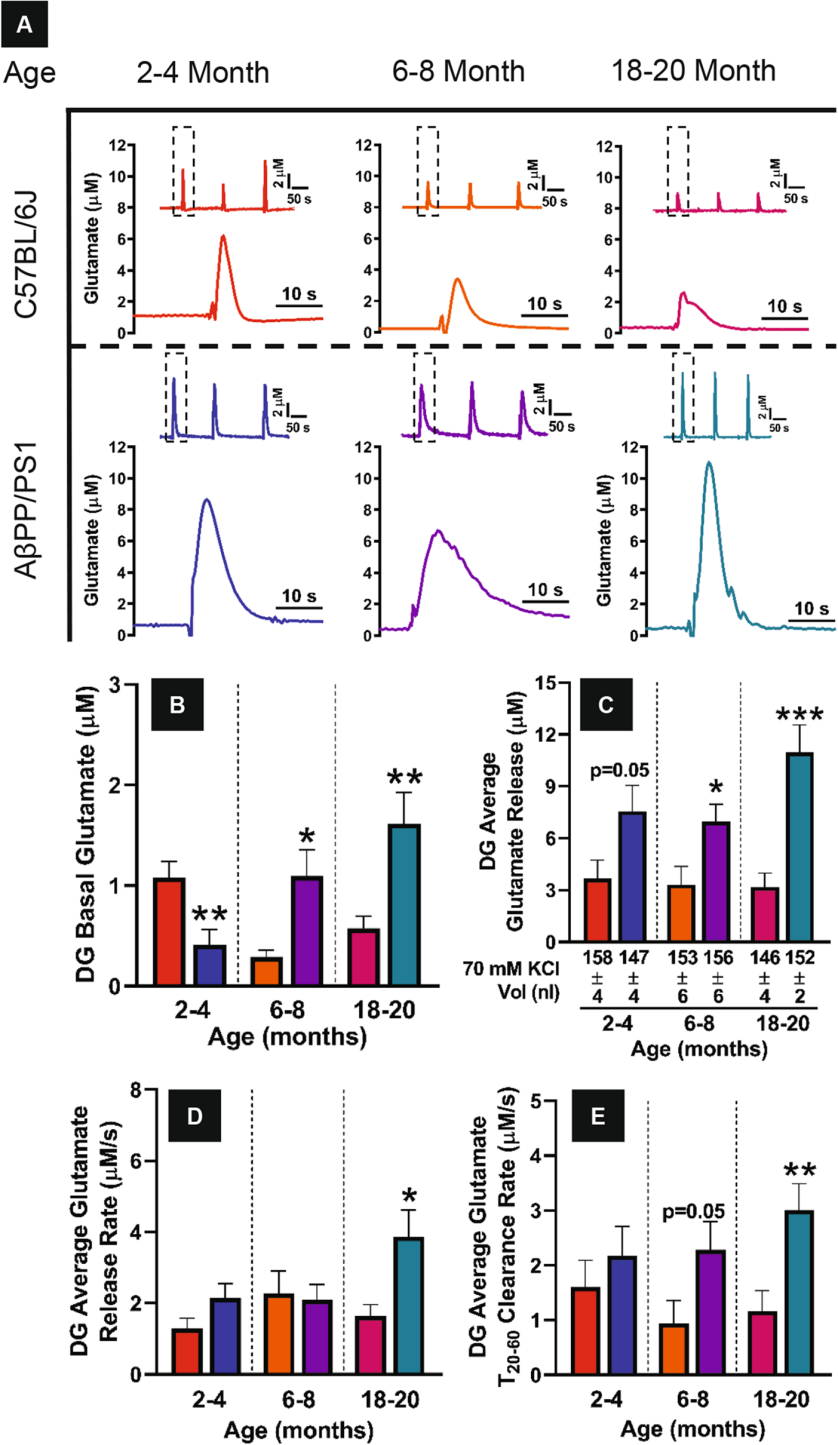
DG glutamate dynamics. (**A**) Representative traces of glutamate release from 70 mM KCl stimulation (**A**). Columns indicate treatment groups while rows indicate hippocampal subfields. The inset trace at the top of each panel depicts the reproducibility of the glutamate signals. The single response shown beneath is a magnified view of the first inset signal (dashed box) designed to give a clearer presentation of glutamate dynamics. Concentration and time axes are consistent in all panels for comparative interpretation. (**B**) Basal glutamate was determined prior to local application of stimulus in each hippocampal subfield. (**C**) The stimulus volume (nl, mean ± SEM) is shown beneath the abscissa. Stimulus volume was monitored and kept consistent across treatment groups for direct comparison of glutamate release. (**D**) Release rate examines the rising portion of the signal while clearance rate (**E**) quantifies the decay portion of the signal. A two-tailed t-test was used to compare genotypes within age groups. *p < 0.05, **p < 0.01, ***p < 0.001, C57BL/6J n = 9–12, AβPP/PS1 n = 9–12.

Representative glutamate release traces from the DG are presented in Fig. 2A for C57BL/6J and AβPP/PS1 mice across all age groups studied. Basal glutamate (Fig. 2B) while decreased at 2–4 months (t_16_ = 3.020, p = 0.008), is elevated at 6–8 months (t_17_ = 2.863, p = 0.01) and further increases by 18–20 months of age (t_22_ = 3.053, p = 0.006) in AβPP/PS1 mice compared to age-matched C57BL/6J. Additionally, C57BL/6J basal glutamate levels are decreased at 6–8 months of age, which contributes to the statistical significance observed in AβPP/PS1 mice at this time point. The average glutamate release from local application of 70 mM KCl was determined by subtracting the peak amplitude from the basal measure prior to stimulus ejection. The maximal amplitude of glutamate release (Fig. 2C) is elevated in AβPP/PS1 at 2–4 (t_19_ = 2.074, p = 0.05) and 6–8 (t_18_ = 2.502, p = 0.02) months of age that continues to increase at the oldest age group studied (t_21_ = 4.308, p = 0.0003) compared to age-matched C57BL/6J mice. The release rate of evoked glutamate is calculated by determining the maximal amplitude of the signal (µM) and dividing by the change in time (s). No differences in release rate (Fig. 2D) are observed between genotypes at 2–4 and 6–8 months of age, but AβPP/PS1 mice release glutamate faster at 18–20 months of age (t_20_ = 2.738, p = 0.01). Clearance of extracellular glutamate was calculated by determining the amplitude change (µM) between 20 and 60 percent of the maximal signal and dividing by the length of time (s) during this signal decay resulting in a clearance rate with units of µM/s. No differences in glutamate clearance (Fig. 2E) are observed at 2–4 months of age, but is elevated in AβPP/PS1 mice at 6–8 (t_18_ = 2.041, p = 0.05), and 18–20 (t_21_ = 2.979, p = 0.007) months of age. In the DG, basal and stimulus-evoked glutamate release increases with disease progression in AβPP/PS1.

### In vivo CA3 glutamate dynamics

**Figure 3:**
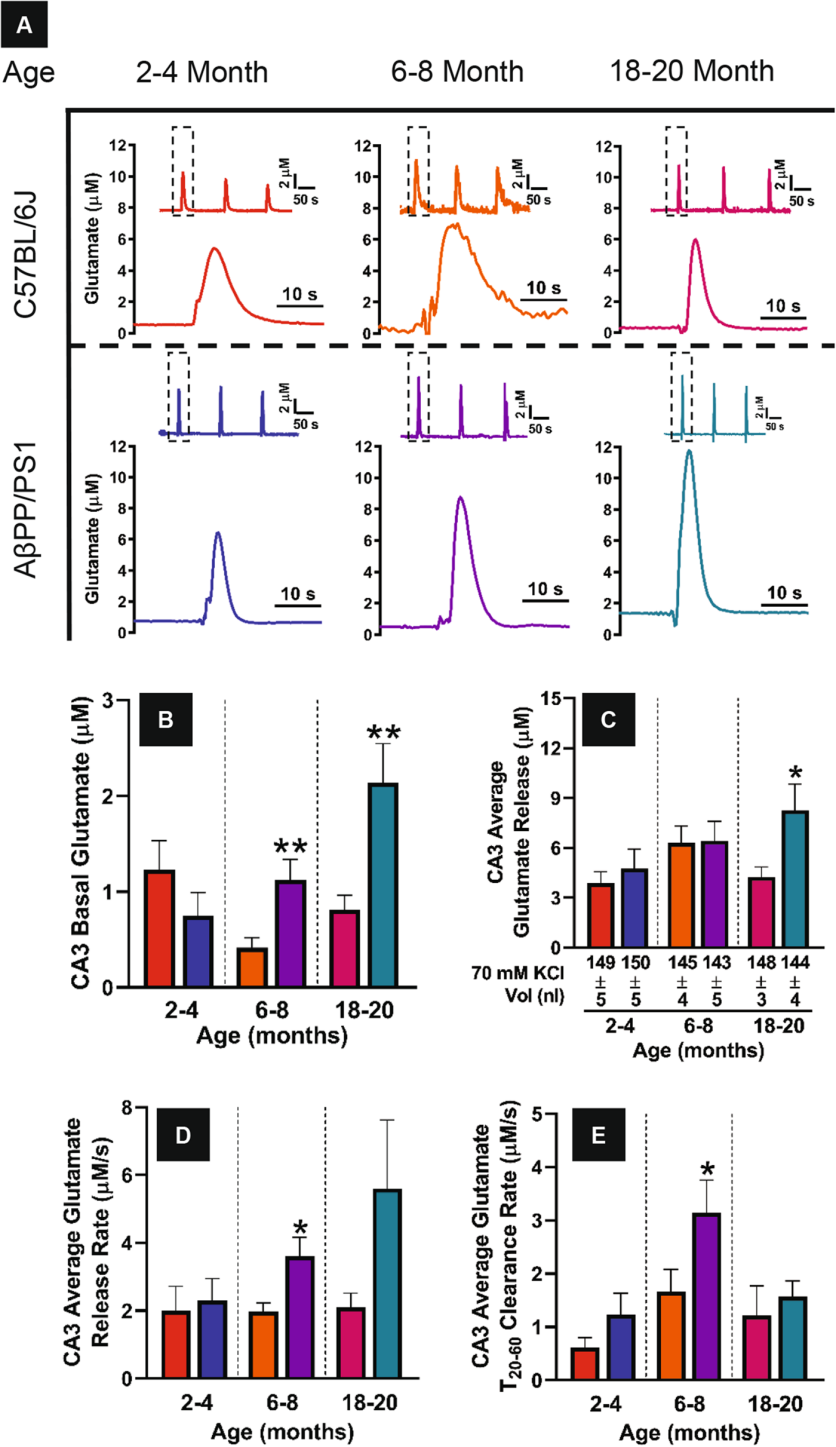
CA3 glutamate dynamics. (**A**) Representative traces of 70 mM KCl evoked glutamate release in the CA3 of 2–4 (left), 6–8 (middle), and 18–20 (right) month old C57BL/6J (top row) and AβPP/PS1 (bottom row) mice. The reproducibility of the glutamate signal (inset) and magnified first signal (dashed box) is shown in each panel. Basal glutamate, stimulus-evoked glutamate release average amplitude, glutamate release rate, and clearance rate (**B**–**E**) were calculated as previously described. A two-tailed t-test was used to compare genotypes within age groups. *p < 0.05, **p < 0.01, C57BL/6J n = 8–12, AβPP/PS1 n = 8–12.

Figure 3A shows representative CA3 glutamate release traces for C57BL/6J and AβPP/PS1 mice across all age groups. No differences in basal glutamate (Fig. 3B) are observed at 2–4 months but becomes elevated in AβPP/PS1 mice at 6–8 months (t_17_ = 3.099, p = 0.007) and continues to increase through 18–20 (t_22_ = 3.074, p = 0.006) months of age. Similar to DG recordings, CA3 basal glutamate levels decrease in 6–8 month-old C57BL/6J mice and contributes to the statistically significant increase observed in AβPP/PS1 mice at this time point. Maximal amplitude of glutamate release (Fig. 3C) is similar across genotypes at 2–4 and 6–8 months of age. CA3 glutamate release is not increased until 18–20 (t_22_ = 2.386, p = 0.03) months of age compared to age-matched C57BL/6J mice. Similar to CA3 basal glutamate, no differences in release rate (Fig. 3D) is observed at 2–4 months, but this becomes elevated at the 6–8 (t_18_ = 2.836, p = 0.01) and 18–20 (t_22_ = 1.691, p = 0.11) months of age in AβPP/PS1 mice. Only the 6–8 month old AβPP/PS1 mice have faster clearance of evoked glutamate (Fig. 3E) compared to age-matched C57BL/6J mice (t_19_ = 2.094, p = 0.04). CA3 basal and evoked glutamate release become elevated as a result of disease progression; similar to the DG but temporally delayed.

### In vivo CA1 glutamate dynamics

**Figure 4:**
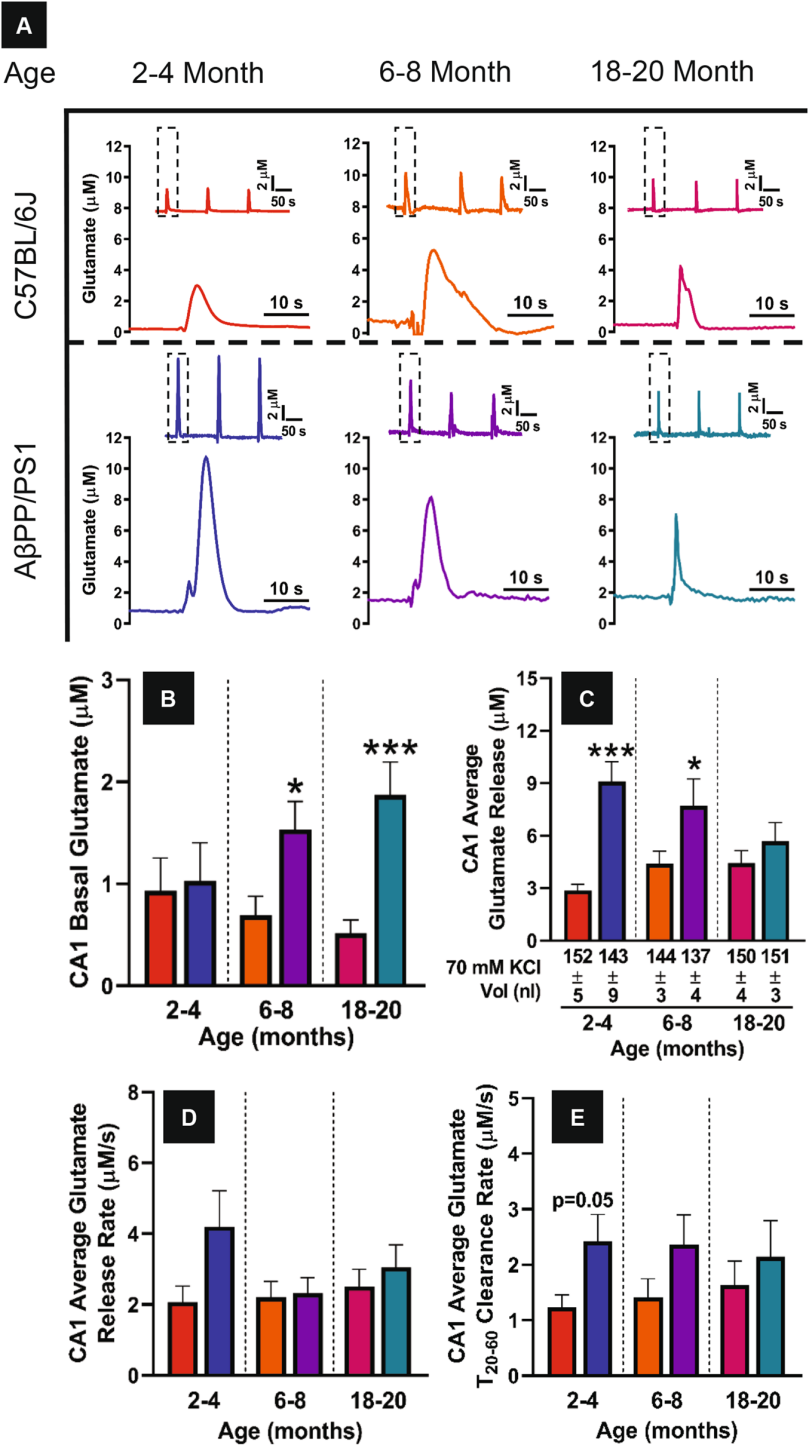
CA1 glutamate dynamics. (**A**) Stimulus-evoked glutamate release representative traces from the CA1 of C57BL/6J and AβPP/PS1 mice (rows) across all ages tested (columns). Within each panel the signal reproducibility is shown as an inset with the corresponding first signal (dashed box) magnified. Basal glutamate, stimulus-evoked glutamate release average amplitude, glutamate release rate, and clearance rate (**B**–**E**) were calculated as previously described. A two-tailed t-test was used to compare genotypes within age groups. *p < 0.05, **p < 0.01, ***p < 0.001, C57BL/6J n = 7–13, AβPP/PS1 n = 9–12.

Figure 4A depicts CA1 representative glutamate release traces for both C57BL/6J and AβPP/PS1 mice across the three age groups tested. At 2–4 months of age no differences in CA1 basal glutamate are observed (Fig. 4B). AβPP/PS1 CA1 basal glutamate increases with disease progression and is elevated at 6–8 (t_20_ = 2.618, p = 0.02) and 18–20 (t_22_ = 3.946, p = 0.001) months of age compared to age-matched C57BL/6J mice. An opposite effect with disease progression is observed with CA1 stimulus-evoked glutamate release (Fig. 4C). The younger age groups studied, 2–4 (t_20_ = 4.834, p = 0.0001) and 6–8 (t_20_ = 2.142, p = 0.04) months of age have elevated glutamate release, but is similar to C57BL/6J mice at 18–20 months of age. AβPP/PS1 CA1 release rate (Fig. 4D) tended to be elevated at the youngest age group only (t_20_ = 1.800, p = 0.08). No differences between genotypes are observed at the other age groups. The clearance of CA1 evoked glutamate (Fig. 4E) is elevated in AβPP/PS1 mice early in disease progression at the 2–4 (t_20_ = 2.078, p = 0.05) and 6–8 (t_20_ 1.612, p = 0.12) month time points, but no difference is observed at 18–20 months of age. CA1 glutamate dynamics are inverted with basal levels increasing and evoked-release decreasing with respect to disease progression.

### Amyloid plaque staining

**Figure 5:**
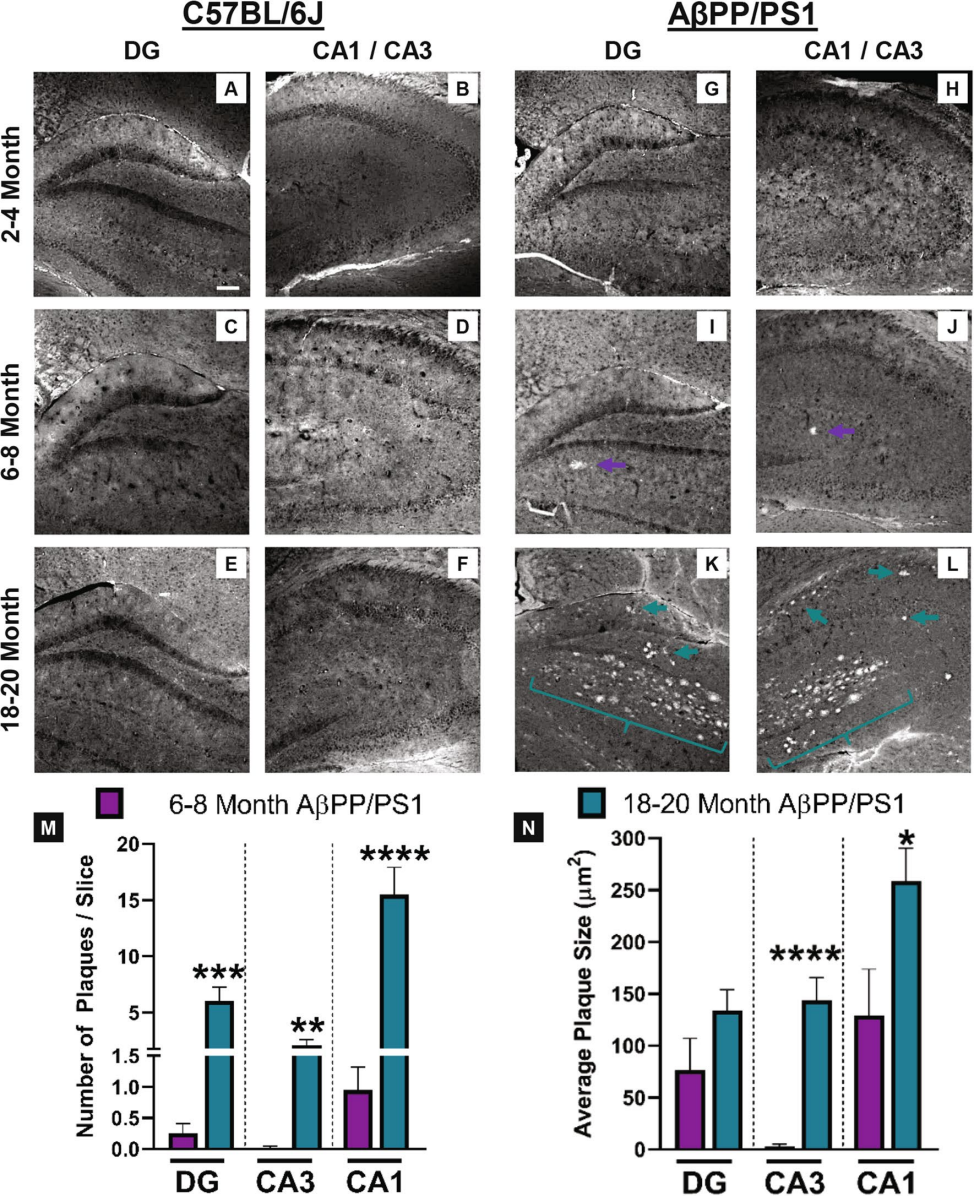
Aβ plaque staining. Representative hippocampal images at 10 × magnification of amyloid plaques staining in C57BL/6J (**A**–**F**) and AβPP/PS1 (**G**–**L**) mice at 2–4, 6–8, and 18–20 months of age. Plaques were only observed in 6–8 month- (**I**,**J**; purple arrows) and 18–20 month-old (**K**,**L**; teal arrows and brackets) AβPP/PS1 mice. Scale bar represents 100 µm. Average number of plaques (**M**) and the average plaque size (**N**) in each hippocampal subregion. A two-tailed t-test was used to compare ages within the AβPP/PS1 mice. *p < 0.05, **p < 0.01, ***p < 0.001, ****p < 0.0001; 6–8 months n = 9–10; 18–20 months n = 11–12 AβPP/PS1 mice.

Amylo-Glo RTD plaque staining reagent was used to measure changes in amyloid plaque pathology throughout the hippocampus. Representative 10 × magnification images form the DG and CA1/CA3 hippocampal subregions from all mice studied are shown in Fig. 5. Plaque pathology was not observed in any C57BL/6J mice nor in the 2–4 month AβPP/PS1 age group as shown in Fig. 5A–H. Amyloid plaques were observed in the 6–8 (Fig. 5I,J) and 18–20 (Fig. 5K,L) month-old AβPP/PS1 mice. As AβPP/PS1 mice aged, the number of amyloid plaques (Fig. 5E) increased in the DG (t_20_ = 4.415; P = 0.0003), CA3 (t_19_ = 3.851; P = 0.001) and CA1 (t_18_ = 5.307; P < 0.0001). Plaque size (Fig. 5F) was also increased with age in the CA3 (t_19_ = 6.069; P < 0.0001) and CA1 (t_18_ = 2.425; P < 0.0260), but not the DG (t_20_ = 1.615; P = 0.12) of AβPP/PS1 mice. Plaque accumulation was observed first in the DG and CA1 of 6–8 month old AβPP/PS1 mice that progressed in quantity and area throughout the hippocampus by the 18–20 month time-point.

## Discussion

One of the first preclinical symptoms associated with AD is attributed to hyperactive hippocampal neuronal networks. Hippocampal hyperactivity is observed in Aβ positive MCI patients, which persists with disease progression despite increasing rates of hippocampal atrophy and dementia scale ratings^9^. Elevated hippocampal activity has also been reported in several mouse models of AD with progressive amyloidosis^24–28^. These changes in neuronal networks are initially observed in mice before plaque deposition^29,30^, supporting a role for soluble Aβ in neuronal hyperactivity. Endogenous Aβ was shown to increase the release probability at excitatory synapses^29,31,32^ as well as stimulate synaptic glutamate release^20,21^. However, as Aβ_42_ accumulation leads to aggregation, the neuronal proximity to the surrounding plaques determines their activity states. Neurons closest to the plaques develop hyperactive phenotypes while those further from plaques are markedly silenced^33^. This may partially be explained by the accumulation of soluble Aβ isoforms surrounding plaques that colocalize with postsynaptic densities and cause synapse loss^34^. Furthermore, this would create a localized area of intense synaptic activity that can propagate Aβ pathology^35,36^ and cause additional plaque deposition^37^. Thus a vicious cycle is created whereby intensified glutamatergic activity and amyloid accumulation extend throughout cortical areas, contribute to seizure activity^38^, and cascade into the degenerative processes observed in AD^39^.

In the present study, learning and memory recall deficits in AβPP/PS1 mice were only observed at the oldest age group studied. Discordant results exist in the literature regarding when AβPP/PS1 mice begin to experience MWM cognitive deficits. Differences in cognition can begin by 6 months of age^40^ with others reporting first appearance at 10 months that progresses with age^41^ that are in line with the present study and our prior work^22^. Increased thigmotaxic behavior was also observed in the oldest AβPP/PS1 age group studied, similar to previous reports in this transgenic AD model^42^. When first navigating the MWM, mice tend to remain close to the wall until they learn to search the middle of the pool for an escape route. While learning the MWM, the thigmotaxic behavior in 18–20 month-old AβPP/PS1 subsided slower and was more prevalent during the probe challenge compared to age-matched C57BL/6J mice. This may be indicative of sensorimotor impairments and anxiety that affected learning and memory in AD mice at 18–20 months of age.

AβPP/PS1 mice progressively accumulate soluble Aβ_42_ starting as early as 3 months of age^43^. This accumulation eventually determines amyloid burden that becomes visible by 6 months of age and increases throughout the lifespan of these transgenic mice^44,45^. Likewise, the present study indicated no plaque deposition at 3 months of age, but by 6 months of age, plaque accumulation was most prominent in the CA1 followed by the DG with little to none observed in the CA3. As AβPP/PS1 mice reach 18 months of age, the magnitude of plaque deposition increases throughout the hippocampus, and this subregion distribution pattern continues including observable plaque pathology in the CA3.

Because plaque burden is a result of Aβ_42_ accumulation in AβPP/PS1 mice^44^, the subregion distribution pattern of plaque deposition is indicative of soluble Aβ_42_ concentration. This disease stage dependent progression of amyloid accumulation and deposition explains the basal and evoked glutamate release data reported in this manuscript. Elevated evoked glutamate release was prominent in the CA1 and DG at 2–4 and 6–8 months of age, but this was not observed until 18–20 months of age in the CA3. Elevated basal glutamate follows a similar hippocampal subregion distribution pattern that becomes more pronounced with age. However, this is temporally delayed to 6–8 months which may indicate amyloid accumulation first sensitizes neurons for increased release, then progresses to consistently elevated circulating levels of glutamate. The CA1 was the only subregion where evoked glutamate release declines with age despite increasing plaque deposition. A reduction of CA1 dendritic architecture was linked to enhanced cellular excitability at an age when plaque deposition was present^26^. However, as Aβ_42_ accumulation progresses, a threshold may be passed where neurons become hypoactive or synapse loss is too pronounced^30^ that diminishes glutamate release. Further studies are required to understand the how hippocampal soluble Aβ_42_ levels contribute to changes in glutamatergic neurotransmission.

The pattern of amyloid deposition presented here is similar to the Braak neuropathological staging of hippocampal amyloid progression in AD^5,6^. This staging also coincides with CA1 neuronal loss occurring before other hippocampal subregions^46,47^. It is known that CA1 neurons are more vulnerable to global cerebral ischemia^48^ and degenerate faster in epileptic patients^49^. The underlying cause of selective CA1 neuronal degeneration is a result of glutamate-mediated excitotoxic mechanisms involving excessive calcium influx through NMDAR activation, mitochondrial dysfunction, and reactive oxygen species. These events culminate in necrotic cell loss that releases more glutamate into the extracellular space thus propagating damage to surrounding neurons. This process supports our discordant CA1 glutamate observations with increasing basal but decreasing evoked glutamate release with age in AβPP/PS1 mice.

This research builds upon a growing body of literature indicating temporally altered hippocampal glutamatergic signaling during the progression of AD pathology. Our previous studies support hippocampal glutamate levels are still elevated at 12 months of age in male AβPP/PS1 mice^22,23^. Others have shown this is not a sex specific characteristic since female AβPP/PS1 mice have elevated CA1 dialysate glutamate levels at 7 months of age that also decline by 17 months of age^41^. Noninvasive techniques such as glutamate chemical exchange saturation transfer GluCEST also indicate a decrease in hippocampal glutamate in 18–20 months old AβPP/PS1 mice^50^. Interestingly these observations are not specific for progression of amyloid pathology. Electrochemical studies in the 5–8 month old tau mouse model of AD, P301L, develop elevated hippocampal glutamate^51,52^. A separate tau mouse model, P301S, also has elevated hippocampal glutamate at 3 months old, that declines at 18–20 months of age^53,54^ as measured by GluCEST techniques. While the amyloid and tau pathology are likely acting through different mechanisms to elicit glutamate release, these studies show a concomitant change in vesicular glutamate transporter 1 that corresponds to the elevated glutamate levels regardless of AD pathology. When considered with the present research, these studies support temporal changes in hippocampal glutamate during AD progression with elevated levels early in pathology that decline in later disease stages. Understanding these types of regional differences may help to refine severity and progression of AD and tailor appropriate treatment options.

In early AD stages, before overt atrophy, overactivation of the NMDAR is hypothesized to impede detection of physiological signals leading to the cognitive impairment observed in AD^39,55^. Accordingly, meta-analysis of memantine treatment, a partial NMDAR antagonist, ameliorates cognitive and functional performance in mild-to-moderate AD patients when administered as monotherapy or in combination with anticholinesterase inhibitors^56^. This treatment only delays cognitive decline and does not have disease modifying benefits^57^. Since memantine modulates glutamate signaling rather than attenuating the glutamatergic tone, the persistently elevated glutamate levels during AD progression may induce excitotoxic effects that accounts for the neuronal, cognitive, and functional loss. As such, drugs that attenuate glutamate release or enhance clearance may provide long-term therapeutic benefits if initiated before signs of cognitive impairment.

## Conclusion

These data support a growing body of literature indicating hyperactive hippocampal glutamate signaling contributes to AD pathogenesis. The temporal hippocampal glutamate changes observed in this study may serve as a biomarker allowing for time point specific therapeutic interventions that can be tailored for maximal efficacy. Simultaneously monitoring changes in hippocampal glutamate with plaque and tangle pathology may further refine stages of AD progression.

## Methods

### Animals

Male C57BL/6J (RRID:IMSR_JAX:000,664) and AβPP/PS1 (RRID:MMRRC_034832-JAX) mice were obtained from Jackson Laboratory (Bar Harbor, ME). Protocols for animal use were approved by the *Laboratory Animal Care and Use Committee* at Southern Illinois University School of Medicine, which is accredited by the Association for Assessment and Accreditation of Laboratory Animal Care. Mice were group housed on a 12:12 h light: dark cycle, and food and water were available ad libitum. Mice arrived to our animal facility at ~ 8 weeks of age and acclimated for 1 week before randomly assigned into one of three age groups: 2–4, 6–8, or 18–20 months for study analysis. Mice were euthanized with an overdose of isoflurane followed by rapid decapitation. Genotypes were confirmed by collecting a 5 mm tail snip for analysis by TransnetYX, Inc (Cordova, TN).

### Chemicals

l-Glutamate oxidase (EC 1.4.3.11) was obtained from Cosmo Bio Co. (Carlsbad, CA) and diluted in distilled, deionized water to make a 1 U/µl stock solution for storage at 4 °C. Sodium phosphate monobasic monohydrate, sodium phosphate dibasic anhydrous, 1,3 phenylenediamine dihydrochloride (mPD), sodium chloride, calcium chloride dihydrate, and H_2_O_2_ (30% in water) were obtained from Thermo Fisher Scientific (Waltham, MA). l-Glutamic acid sodium salt, bovine serum albumin (BSA), glutaraldehyde, KCl, dopamine hydrochloride (DA), and l-ascorbic acid (AA) were obtained from Sigma-Aldrich Co. (St. Louis, MO).

### Morris water maze training and probe challenge

The MWM was used to assess spatial learning and memory recall. Mice were trained to utilize visual cues placed around the room to repeatedly swim to a static, hidden escape platform (submerged 1 cm below the opaque water surface) regardless of starting quadrant^23,58^. The MWM paradigm consisted of 5 consecutive training days with three, 90 s trials/day and a minimum inter-trial-interval of 20 min. Starting quadrant was randomized for each trial. After two days without testing, the escape platform was removed and all mice entered the pool of water from the same starting position for a single, 60 s probe challenge to test long-term memory recall. The ANY-maze video tracking system (Stoelting Co., Wood Dale, IL; RRID:SCR_014289) was used to record mouse navigation during the training and probe challenge. The three trials for each training day were averaged for each mouse.

### Enzyme-based microelectrode arrays

**Figure 6:**
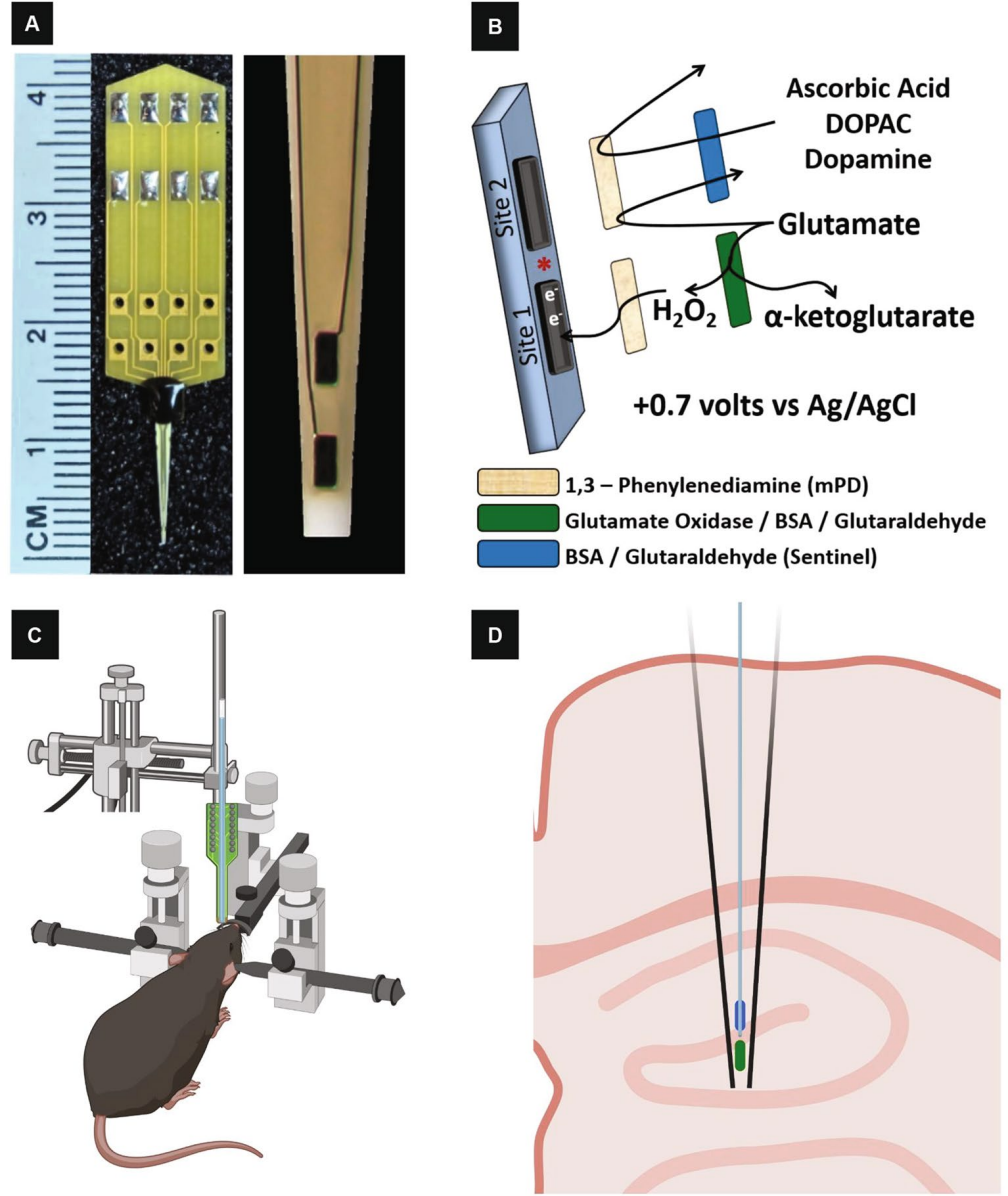
Images, enzyme coating, and implantation of MEAs for selective glutamate recordings. MEA images are shown in (**A**). The R2 MEA consists of a printed circuit board (yellow, top) with soldered gold plated pins for connection to the FAST system and a wire bonded working electrode (bottom, tapered tip) that measures 4 cm in length. A magnified view of the working electrode with two independent platinum recording sites (50 × 100 microns) is shown on the right. A coating schematic illustration for selective glutamate recordings is shown (**B**). Site 1 is coated with glutamate oxidase, BSA, and glutaraldehyde (green) while Site 2 is coated with the inactive protein matrix consisting of BSA and glutaraldehyde (blue). Glutamate is enzymatically cleaved by glutamate oxidase to form the electroactive reporter molecule H_2_O_2_, but not on Site 2 coated with the inactive protein matrix. MEA selectivity for glutamate is improved by electropolymerizing mPD onto both recording surfaces, which forms a size exclusion layer that blocks ascorbic acid, 3,4-dihydroxyphenylacetic acid (DOPAC), and DA that are electrochemically active at our working potential. Mice are isoflurane anesthetized and placed in a stereotaxic frame (**C**). The MEA (green)/micropipette assembly (blue) is fixed to probe holder (**C**) attached to the stereotaxic arm for positioning into the different hippocampal subregions (**D**). Images (**C**) and (**D**) were prepared using BioRender.

Enzyme-based MEAs with platinum (Pt) recording surfaces (Fig. 6A) were fabricated, assembled, coated (Fig. 6B), and calibrated for in vivo mouse glutamate measurements as previously described^59–61^. One microliter of glutamate oxidase stock solution (1 U/µl) was added to 9 µl of a 1.0% BSA and 0.125% glutaraldehyde w/v solution and applied dropwise to a Pt recording surface. This preparation aides in enzyme adhesion to the Pt recording surface for enzymatic degradation of glutamate to α-ketoglutarate and H_2_O_2_, the electroactive reporter molecule. The other Pt recording site (self-referencing or sentinel site) was coated with the BSA/glutaraldehyde solution, which is unable to enzymatically generate H_2_O_2_ from l-glutamate. A potential of + 0.7 V vs a Ag/AgCl reference electrode was applied to the Pt recording surfaces resulting in oxidation of H_2_O_2_. While + 0.7 V is capable of oxidizing potential interferants, such as AA and DA, lower potentials are unable to adequately oxidize H_2_O_2_ and subsequently detect glutamate^62^. The current generated from the two electron transfer was amplified and digitized by the Fast Analytical Sensing Technology (FAST) 16mkIII (Quanteon, LLC; Nicholasville, KY) electrochemistry instrument.

### mPD electropolymerization

After enzyme coating, Pt recording surfaces were electroplated with 5 mM mPD in 0.05 M phosphate buffered saline (PBS) to block potential interferants oxidizable at + 0.7 V (Fig. 6B). FAST electroplating software applied a triangular wave potential with an offset of − 0.5 V, peak-to-peak amplitude of 0.25 V, at a frequency of 0.05 Hz, for 20 min. This created a size exclusion layer that restricts the passage of AA, DA, uric acid and 3,4-dihydroxyphenylacetic acid to the Pt recording surface^63^.

### Calibration

MEAs were calibrated according to previously published protocols^22,23^. MEAs were placed in a stirred solution of 40.0 mL of 0.05 M PBS held constant at 37 °C using a recirculating water bath (Stryker Corp., Kalamazoo, MI). Using a glass Ag/AgCl reference electrode (Bioanalytical Systems, Inc., West Lafayette, IN), MEAs were calibrated with final beaker concentrations of 250 µM AA, 10, 20, 30, and 40 µM l-glutamate, 2 µM DA, and 8.8 µM H_2_O_2_ to create a standard curve for the conversion of current to glutamate concentration. Seventy-one MEAs were calibrated and the average ± standard error of the mean (SEM) for glutamate sensitivity was 8.9 ± 0.1 pA/μM (R^2^ = 0.996 ± 0.001), selectivity ratio of 442 ± 63 to 1, and limit of detection of 0.23 ± 0.03 μM based on a signal-to-noise ratio of 3.

### Microelectrode array/micropipette assembly

Glass micropipettes (1.0 mm outer diameter, 0.58 mm internal diameter; World Precision Instruments, Inc., Sarasota, FL) were pulled using a vertical micropipette puller (Sutter Instrument Co., Novato, CA). The tip was “bumped” to create an internal diameter of 12–15 µm. The micropipette tip was positioned between the pair of recording sites and mounted 100 µm above the MEA surface (Fig. 6D). The micropipettes were filled with sterile filtered (0.20 µm) 70 mM KCl (70 mM KCl, 79 mM NaCl and 2.5 mM CaCl_2_, pH 7.4). Fluid was pressure-ejected from the glass micropipette using a Picospritzer III (Parker-Hannafin, Cleveland, OH), with pressure (5–15 psi) adjusted to consistently deliver volumes between 100–200 nl over 1–2 s intervals. Ejection volumes were monitored with a stereomicroscope (Luxo Corp., Elmsford, NY) fitted with a calibrated reticule^20^.

### Reference electrode

A Ag/AgCl reference wire was prepared as previously described^22,23^. The Teflon from both ends of a silver wire (200 μm bare, 275 μm coated; A-M Systems, Carlsberg, WA) was removed and one end soldered to a gold-plated connector (Newark element14 Chicago, IL). The other stripped end was placed (cathode) into a 1 M HCl bath saturated with NaCl that also contained a stainless steel counter wire (anode). Passing a + 9 V DC to the cathode *versus* the anode for 15 min deposits Ag/Cl onto the stripped wire.

### In vivo anesthetized recordings

One week after MWM, mice were anesthetized using 1.5–2.0% isoflurane (Abbott Lab, North Chicago, IL) in a calibrated vaporizer (Vaporizer Sales & Service, Inc., Rockmart, GA)^63^. The mouse was placed in a stereotaxic frame fitted with an anesthesia mask (Fig. 6C; David Kopf Instruments, Tujunga, CA). Body temperature was maintained at 37 °C with a water pad (Braintree Scientific Inc., Braintree, MA) connected to a recirculating water bath. A craniotomy was performed to access the DG (AP: − 2.0, ML: ± 1.0, DV: − 2.2 mm), CA3 (AP: − 2.0, ML: ± 2.0, DV: − 2.2 mm), and CA1 (AP: − 2.0, ML: ± 1.0, DV: − 1.7 mm) from Bregma^64^. Recordings were conducted using a two electrode system whereby a Ag/AgCl reference wire was positioned beneath the skull and rostral to the craniotomy and a working electrode was positioned in one of the hippocampal subregions (Fig. 6D). Constant voltage amperometry (4 Hz) was performed with a potential of + 0.7 V vs the Ag/AgCl reference electrode applied by the FAST16mkIII. MEAs reached a stable baseline for 60 min before a 10 s basal glutamate determination and pressure ejection studies commenced. Once five reproducible signals were evoked, the MEA was repositioned into a new hippocampal subfield, which was randomized for each mouse. The FAST software saved amperometric data, time, and pressure ejection events. Calibration data, in conjunction with a MATLAB (MathWorks, Natick, MA; RRID:SCR_001622) graphic user interface program was used to calculate basal, stimulus-evoked, and clearance of extracellular glutamate. The evoked glutamate signals in each hippocampal subfield were averaged into a representative signal for comparison.

### Amyloid plaque staining and semi-quantification

Sections were prepared, stained and quantified as previously described^22,23^. After electrochemistry, brains were removed and post-fixed in 4% paraformaldehyde for 48 h and then transferred into 30% sucrose in 0.1 M phosphate buffer for at least 24 h prior to sectioning. Twenty µm coronal sections through the hippocampus were obtained using a cryostat (Model HM525 NX, Thermo Fisher Scientific). Mounted sections were treated with 10% H_2_O_2_ in 20% methanol for 10 min, transferred to 70% ethanol solution for 5 min, and then washed with PBS for 2 min. Sections were incubated for 10 min in Amylo-Glo RTD (1:100; Biosensis, Temecula, CA), submerged in physiological saline for 5 min, and rinsed three times in separate PBS solutions for 2 min. Sections were coverslipped using Fluoromount-G (SouthernBiotech; Birmingham, AL). Staining intensity was controlled for by imaging all sections the next day. Images were captured with an Olympus 1 × 71 microscope equipped with an Olympus-DP73 video camera system, and a Dell Optiplex 7020 computer. National Institutes of Health Image J Software (v. 1.48; RRID:SCR_003070) was used to measure relative staining density by using a 0–256 Gy scale. Staining density was obtained when background staining was subtracted from mean staining intensities on every sixth section through the hippocampus. Individual templates for the DG, CA3, and CA1 were created and used on all brains similarly. Measurements were performed blinded, and approximately four sections were averaged to obtain one value per subject. Amyloid plaques were identified by a dense spherical core of intense staining that were often surrounded by a less compact spherical halo.

### Data analysis

Prism (GraphPad Prism 8 Software, Inc., La Jolla, CA; RRID:SCR_002798) software was used for statistical analyses. For glutamate measurements and amyloid plaque staining, hippocampal subregions were examined independently. For statistical analysis, genotypes were compared within age groups and all tests are listed in the figure legends. Outliers were identified with a single Grubbs’ test (alpha = 0.05) per group. Data are represented as mean ± SEM and statistical significance was defined as p < 0.05.

## Data Availability

Data is available upon reasonable request.

## Abbreviations

mPD: 1,3-Phenylenediamine dihydrochloride
AUC: Area under the curve
Aβ: β-Amyloid
AD: Alzheimer’s disease
ANOVA: Analysis of variance
AP: Anterior/posterior
AA: Ascorbic acid
BSA: Bovine serum albumin
DG: Dentate
DA: Dopamine
DV: Dorsal/ventral
FAST: Fast analytical sensing technology
GluCEST: Glutamate chemical exchange saturation transfer
ML: Medial/lateral
MEA: Microelectrode array
MCI: Mild cognitive impairment
MWM: Morris water maze
NMDAR: N-methyl-d-aspartate receptors
PBS: Phosphate buffered saline
